# Full-length HIV-1 immunogens induce greater T lymphocyte responses to conserved epitopes than conserved-region-only HIV-1 immunogens in monkeys

**DOI:** 10.1186/1742-4690-9-S2-O70

**Published:** 2012-09-13

**Authors:** KE Stephenson, A SanMiguel, NL Simmons, K Smith, JJ Szinger, BT Korber, DH Barouch

**Affiliations:** 1 Beth Israel Deaconess Medical Center and Harvard University, Boston, MA, USA; 2 Los Alamos National Laboratory, Los Alamos, NM, USA

## Background

A global HIV-1 vaccine will need to induce broadly reactive immune responses against conserved HIV-1 regions. It is currently unclear how best to elicit these responses by vaccination. We therefore compared the immunogenicity of a bivalent full-length HIV-1 Gag/Pol/Env mosaic vaccine, a trivalent full-length HIV-1 Gag/Pol/Env mosaic vaccine, and a bivalent mosaic vaccine containing only conserved HIV-1 Gag/Pol/Env epitopes in rhesus monkeys.

## Methods

We immunized 18 rhesus monkeys with rAd35 (prime) and rAd26 (boost) vectors expressing bivalent full-length (N=6), trivalent full-length (N=6), or bivalent conserved-region-only (N=6) HIV-1 Gag/Pol/Env mosaic immunogens. We assessed HIV-1-specific and conserved-region-specific cellular immune responses by ELISPOT using global PTE and vaccine-matched peptides. Responses were mapped to individual epitopes and were identified as CD4+ or CD8+ through cell-depletion assays. Comparisons were performed by Wilcoxon rank-sum tests.

## Results

There was no difference in the breadth of HIV-1-specific T lymphocyte responses elicited by the bivalent and trivalent full-length mosaic vaccines (P=.686). However, the bivalent full-length vaccine generated a greater breadth of HIV-1-specific CD8+ T lymphocyte responses than the conserved-region-only vaccine (P=.007). The bivalent full-length vaccine also generated equivalent breadth of CD8+ T lymphocyte responses to conserved HIV-1 epitopes compared to the conserved-region-only vaccine (P=1.000), and surprisingly, the responses generated by the full-length vaccine to conserved HIV-1 epitopes were greater in magnitude than those generated by the conserved-region-only vaccine (P=.008).

## Conclusion

These data demonstrate that an HIV-1 mosaic vaccine expressing full-length antigens elicited greater responses to conserved epitopes than a mosaic vaccine expressing only concatenated conserved HIV-1 regions. In addition, the bivalent and trivalent full-length mosaic vaccines generated comparable breadth of HIV-1-specific CD8+ T lymphocyte responses. These results support the clinical development of the bivalent full-length HIV-1 mosaic vaccine.

